# Gene Expression Analysis Reveals Age and Ethnicity Signatures Between Young and Old Adults in Human PBMC

**DOI:** 10.3389/fragi.2021.797040

**Published:** 2022-02-03

**Authors:** Yang Hu, Yudai Xu, Lipeng Mao, Wen Lei, Jian Xiang, Lijuan Gao, Junxing Jiang, Li`an Huang, Oscar Junhong Luo, Jinhai Duan, Guobing Chen

**Affiliations:** ^1^ Institute of Geriatric Immunology, Department of Microbiology and Immunology, School of Medicine, Jinan University, Guangzhou, China; ^2^ NHC Key Laboratory of Male Reproduction and Genetics, Guangdong Provincial Reproductive Science Institute, Guangdong Provincial Fertility Hospital, Guangzhou, China; ^3^ Department of Systems Biomedical Sciences, School of Medicine, Jinan University, Guangzhou, China; ^4^ Nanfang Hospital, Southern Medical University, Guangzhou, China; ^5^ Department of Neurology, the First Affiliated Hospital, Jinan University, Guangzhou, China; ^6^ Eastern Department of Neurology of Guangdong General Hospital, Guangdong Academy of Medical Sciences, Guandong, China

**Keywords:** immune aging, race and ethnicity, PBMC, WGCNA, biomarkers

## Abstract

Human immune system functions over an entire lifetime, yet how and why the immune system becomes less effective with age are not well understood. Here, we characterize peripheral blood mononuclear cell transcriptome from 132 healthy adults with 21–90 years of age using the weighted gene correlation network analyses. In our study, 113 Caucasian from the 10KIP database and RNA-seq data of 19 Asian (Chinese) are used to explore the differential co-expression genes in PBMC aging. These two dataset reveal a set of insightful gene expression modules and representative gene biomarkers for human immune system aging from Asian and Caucasian ancestry, respectively. Among them, the aging-specific modules may show an age-related gene expression variation spike around early-seventies. In addition, we find the top hub genes including NUDT7, CLPB, OXNAD1, and MLLT3 are shared between Asian and Caucasian aging related modules and further validated in human PBMCs from different age groups. Overall, the impact of age and race on transcriptional variation elucidated from this study may provide insights into the transcriptional driver of immune aging.

## Introduction

Aging is a multifaceted process, involving numerous molecular and cellular mechanisms in the context of different organ systems (Lopez-Otin et al., 2013). A crucial component of aging is a set of functional and structural alterations in the immune system that can diminish the effectiveness of vaccinations, increase disease susceptibility, and contribute to mortality in older adults (Nikolich-Zugich, 2018). In addition to alterations in the stromal microenvironment in primary and secondary lymphoid organs, cell-intrinsic changes like cell numbers, ratio, and function in both innate and adaptive immune cells play an important role in age-associated immune dysfunction. These alterations and transformations manifest themselves in increased morbidity and mortality of older organisms. However, the interplay between the PBMC age-related gene expression changes that affect the immune aging remains incompletely elucidated, and there is no clear understanding of which gene changes are primary, arising as a consequence of aging, and which may be secondary, adaptive or compensatory to the primary changes. Thus, transcriptome analyses may lend greater insight than a static genetic investigation. In contrast to the relatively invariable genome sequence, the transcriptome is highly dynamic and changes in response to stimuli. Therefore, the aim of this study is to exploit a large-scale population-based strategy to systematically identify genes and pathways differentially expressed as a function of chronological age.

More importantly, analyses of human blood samples from different race and ethnicity uncover significant aging-related variations in various subsets of PBMCs (Noren Hooten et al., 2018). For example, a study on PBMC subsets characterizes not only the presence of benign ethnic neutropenia among African Americans but further discovers a higher proportion of CD19^+^ cells and a lower proportion of CD3^+^ cells than in Caucasian population (Tollerud et al., 1989). Moreover, the proportions of PBMCs’ subpopulation in Asian cohorts are also different. Choong and colleagues observe that there are differences in cell counts for T, NK, and CD4^+^ cells as well as in the CD4/CD8 ratio among healthy Malaysians, Chinese, and Indians across the life span (18–71 years) (Choong et al., 1995). In addition, Indians are significantly different from Malays and Chinese. Indians have higher T cells, higher CD4 cells, higher CD4/CD8 ratio, and lower NK cells; Chinese donors have lower B-cell levels than Malays and Indians (Choong et al., 1995). Recent studies also have shown that there are differences in gene expression among European-derived and Asian-derived populations due to the common genetic variants in Epstein-Barr virus (EBV)-transformed lymphoblastoid cell lines (Spielman et al., 2007). Despite the importance of age and race in shaping immune cell numbers and functions, it is not known whether some similarities or differences of gene expression changes exist in Asian and Caucasian immune systems across the life span.

To study this, we analysed the PBMC transcriptomes of 19 healthy Asian from our own lab and 113 Caucasian adults at different aging-stage from the 10KIP (10KIP, https://comphealth.ucsf.edu/app/10kimmunomes) by WGCNA method, respectively. WGCNA and differential gene expression analysis were used to obtain differential co-expression genes. We further explored immune system aging signatures in Asian and Caucasian through protein-protein interaction (PPI) and functional enrichment analysis combined with qPCR analysis. The study provided a potential basis to understand in which ways aging differentially affected the immune systems between ethnicities and discovered a common genetic variant that greatly impacted normal PBMC aging.

## Materials and Methods

**FIGURE 1:**
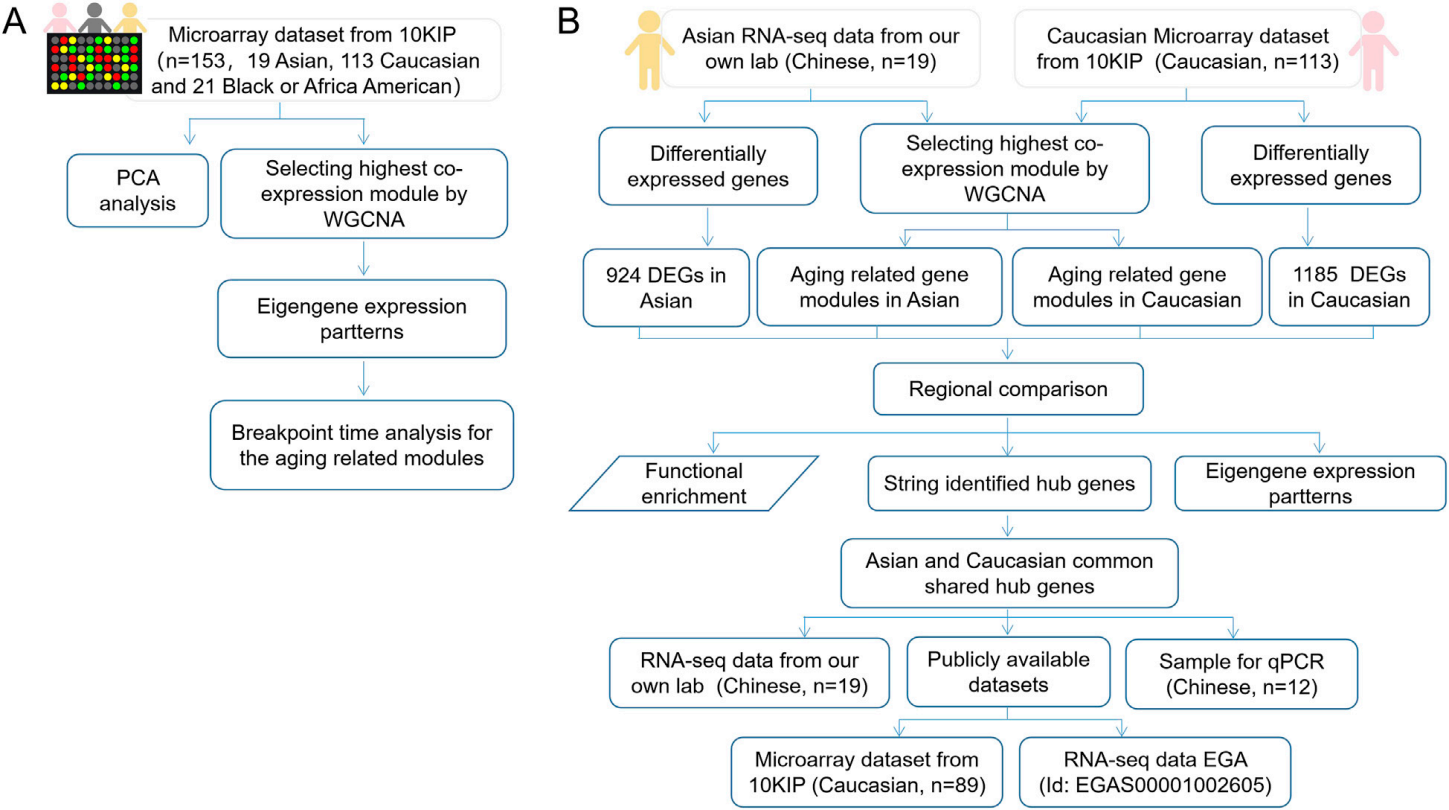
The flow chart of overall study. **(A)** A total analysis of 153 healthy human subjects in 10KIP. **(B)** The anlysis of 133 Caucasian individuals and 19 Asian (Chinese) individuals.

The flow chart of the overall study was shown in Figure 1. We elaborated on each step in the following sub-sections.

### Human Subjects

All studies were conducted following approval by the Ethics Committee of Jinan University (Approval #:KY-2020–027). Following informed consent, blood samples were obtained from 31 healthy volunteers residing in the Guangzhou, China region recruited by the First affiliated Hospital of Jinan University and Guangzhou First People’s Hospital. For selecting the older adults 65 years and older, the eligibility criteria were in line with the 2019 NIH Policy on Inclusion Across the Lifespan (NOT-OD-18–116) (Kuchel, 2019). Subjects were carefully screened and excluded if undergoing potentially a history of diseases and medications, as well as frailty according to the eligibility criteria (Kuchel, 2019). Besides, the donors who had smoking history or quit smoking <2 years were also excluded in our study and all donors were asked for consent for genetic research. Fresh blood was collected into heparin tubes and PBMCs were isolated by density gradient centrifugation using Ficoll-Paque Plus (GE) and washed with Ca/Mg-free PBS twice.

### Ribonucleic Acid-Seq Library Generation and Processing

A total amount of 1 µg RNA per sample, isolated from PBMCs using the TRIzol (Invitrogen, United States), was used as input material for the RNA-seq sample preparations. Sequencing libraries were constructed using NEBNext^®^ UltraTM RNA Library Prep Kit for Illumina^®^ (NEB, United States) following manufacturer’s protocols. Final libraries were assessed on a Bioanalyzer DNA High Sensitivity Chip (Agilent Technologies). Paired-end sequencing (2 × 150 bp) of stranded total RNA libraries was carried out in Illumina NovaSeq 6,000. The FASTQC tool was used to assessed the quality of the raw sequencing data, which computed read quality using summary of per-base quality defined using the probability of an incorrect base call. According to our quality criteria, reads with more than 30% of their nucleotides with a Phred score under 30 were removed, whereas samples with more than 20% of such low-quality reads were dropped from analyses. Reads from samples that passed the quality criteria were quality-trimmed and filtered using trimmomatic. High-quality reads were then used to estimate transcript abundance using RSEM. Finally, the estimate transcript abundance (read counts) was renormalized to include only protein-coding genes and all the downstream analyses were based on high quality data.

### Microarray Data Acquisition

The microarray-based expression from 10 KIP provided by Lu *et al.* (Zalocusky et al., 2018) was downloaded from the 10,000 immunomes project (10KIP, https://comphealth.ucsf.edu/app/10kimmunomes). This dataset contained quantile normalized genome-wide expression profiles of 153 adult human PBMC samples from young to old adults, including samples from 65 young (ages 21–40: 24 men, 41 women), 40 middle-aged (ages 41–64: 18 men, 22 women), and 48 older subjects (65+: 20 men, 28 women) and containing three races including 19 Asian, 113 Caucasian, and 21 Black or Africa American.

### Identification of Key Co-Expression Modules Using WGCNA

WGCNA R software package was applied to identify the co-expression modules of highly correlated genes among samples and related modules to external sample traits (Langfelder and Horvath, 2008). A more detailed description of the WGCNA method was described in our previous study (Hu et al., 2018). In brief, sample was clustered to recognize and remove outlier samples by the average linkage method. Then, the optimum soft thresholding power (β) was selected to obtain a scale-free topology fitting index of >0.8. The soft thresholding power *β* = 6 was used in the analysis of 153 microarray data set. Similarly, the soft thresholding power *β* = 5 was in 113 Caucasian data set and the power *β* = 6 in 19 Asian (Chinese). By using the soft thresholding power (β), the topological overlap matrix (TOM) and the corresponding dissimilarity matrix (1-TOM) were calculated, which was further used to classify the similar gene expressions into different gene co-expression modules (Langfelder and Horvath, 2008). Afterwards, highly similar dynamic modules were merged into larger ones at the cutline of 0.2. The module eigengene (ME) was the first principal component of the expression matrix which represented the gene expression profile of the entire module. Afterwards, the correlation between MEs and previously sample traits was assessed to identify the most relevant clinically significant module by Pearson’s correlation analysis. Meanwhile, the most significant module was also validated by calculating the gene significance (GS) and module membership (MM) (Langfelder and Horvath, 2008). In our study, the gene expression data profiles of microarray data and RNA-seq profile were collected separately to construct gene co-expression networks by using the WGCNA package.

### Screening the Differentially Expressed Genes and Comparing With the Gene Modules of Interest

In order to find the differentially expressed genes (DEGs) between Asian and Caucasian, “limma” R package was applied in the Asian RNA-seq and Caucasian dataset to screen out DEGs, with the cut-off criteria of |logFC| ≥ 0.50 and adjusted *p* < 0.05 (Ritchie et al., 2015). The DEGs of the Asian and Caucasian dataset were visualized by a volcano plot. Subsequently, the overlapping genes between DEGs and co-expression modules, listed by the Venn diagram using the R package VennDiagram, were used to identify potential prognostic genes (Chen and Boutros, 2011). Benjamini-Hochberg *p*-value correction was used to select differentially expressed genes at an FDR adjusted *p*-value of 5%.

### Functional Annotation for the Modules of Interest

For genes in each module, Gene Ontology (GO) and Kyoto Encyclopedia of Genes and Genomes (KEGG) enrichment analyses were conducted to analyze the biological functions of gene modules. The *clusterProfiler* package offered a gene classification method, namely, groupGO, to classify genes based on their projection at a specific level of the GO corpus, and provided functions, *enrichGO* and *enrichKEGG*, to calculate enrichment test for GO terms and KEGG pathways based on hypergeometric distribution (Yu et al., 2012). In our study, *clusterProfile*r package was used to perform the gene ontology (GO) and KEGG pathway enrichment analysis. Significant GO terms were defined with an adjusted *p* < 0.05 and count >6. For the KEGG pathway analysis, the *enrichKEGG* function was utilized and adjusted *p* < 0.01 was set as a cutoff. We used the Benjamini-Hochberg FDR multiple test correction to assess significance of hypergeometric *p*-values.

### Network Analysis of Module Genes/Hub Genes

The hub genes were identified based on protein interaction evidence from the STRING database (version 11.0; http://string-db.org/) (Szklarczyk et al., 2019). The evidence of protein interaction network for key modules from the STRING database was retrieved by an interaction score with highest confidence (0.9). The Cytoscape plugin cytoHubba was used to rank nodes (genes) based on the Maximal Clique Centrality (MCC) topological method (Chin et al., 2014), and the top-5 genes were selected as hub genes for verification. MCC assumes that the node network is an undirected network; given a node *v*, S(*v*) is the set of the maximal cliques containing *v*, and (| C | −1)! is the product of all positive integers less than | C |. The calculation is as follows:

MCC(v)=∑C∈S(v)(|C|−1)!

### Validation of the Hub Genes

In order to confirm the reliability of the hub genes, we tested the expression patterns of the hub genes from healthy individuals including 7 young (ages: 23–30) and 5 old (ages: ≧74). The expression level of each hub gene between young and old individuals was plotted as a violin graph. Total RNA from PBMCs was extracted by TRIzol (Invitrogen, United States). Synthesis of cDNA was performed by using 1 μg of total RNA with PrimeScriptTM Reverse transcriptase (Takara) according to the manufacturer’s instructions. Specific primers used for qPCR were listed in the Supplementary Table S14. ACTB (NM_001,101.5) was used as a reference gene for normalization. Quantitative real-time PCR was performed using the SYBR^®^ Premix Ex Taq Kit (Takara) in a CFX96 Real Time PCR System (Bio-Rad Laboratories, Hercules, CA, United States) for at least three independent experiments. The relative gene expression levels were normalized to ACTB (NM_001,101.5) and quantified using the 2-^ΔΔCT^ method.

## Results

### Aging and Race Cause Transcriptomic Variations Over Human Adult Lifespan

**FIGURE 2:**
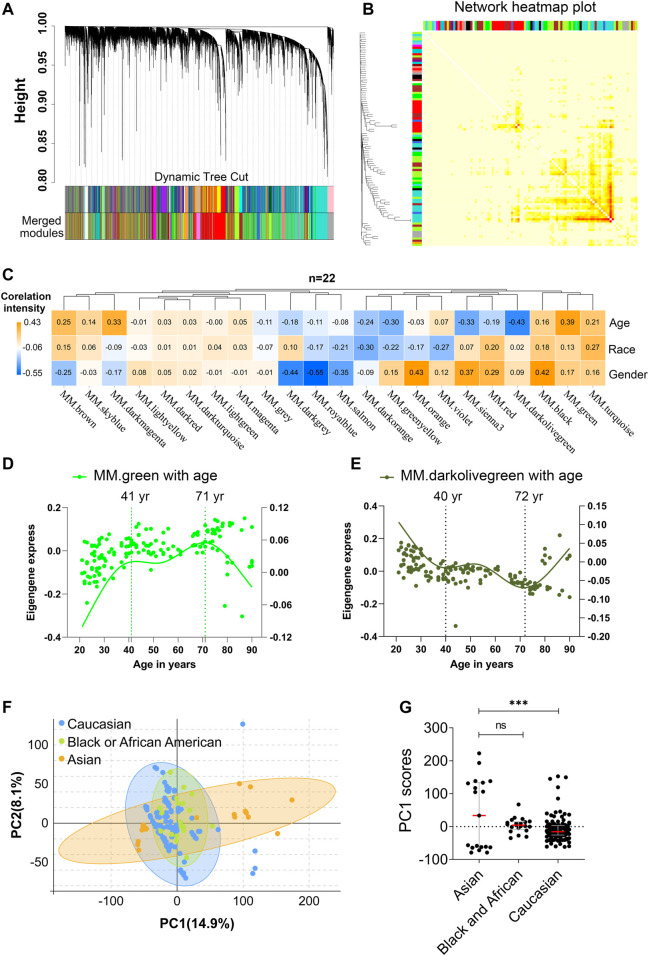
Age and race influenced the transcriptomic variations over human adult lifespan. WGCNA approach was applied for gene module construction for the transcriptome data of 153 healthy human subjects in 10KIP. Principal component analyses (PCA) were calculated individually. **(A)** Cluster dendrogram. Each branch represented one gene, and each color below denoted one co-expression gene module. The two colored rows below the dendrogram represented the original and merged modules, respectively. **(B)** Eigengene adjacency heatmap of different modules. Each module showed independent validation to each other, and higher correlation indicated higher co-expression interconnectedness. **(C)** Heatmap of the Pearson’s correlation coefficient between trait (age, race and gender) and module eigengenes (ME, *n* = 22). The column and row corresponded to ME and trait, respectively. Each cell contained the value of Pearson’s correlation coefficient. The table was color-coded by correlation according to the color legend. The *p*-value < 0.05 represented statistical significance. **(D,E)** The characteristic gene expression changed during PBMC aging. The left and right-hand *Y*-axis represented the eigengene expression of each module and trend line for each individuals, respectively. **(F,G)** Principal component1 scores (PC1) were calculated for each individual from principal component analyses (PCA). PC1 scores from transcriptomic data were differentially expressed among different races. Wilcoxon rank-sum test was used to compare data from Asian (*n* = 19) and Caucasian (*n* = 113) or African American subjects. Dot plot represented median and IQR values; ****p < 0.0001, ***p < 0.001, **p < 0.01, *p < 0.05, n. s.: non-significant.

To identify main factors of variation in PBMC aging transcriptomic data, the microarray-based gene expression from 10 KIP provided by Lu *et al.* (Zalocusky et al., 2018) was downloaded from the 10,000 immunomes project (10KIP, https://comphealth.ucsf.edu/app/10kimmunomes). This dataset contained quantile normalized genome-wide expression profiles of 153 adult human PBMC samples from young to old adults, including samples from 65 young (ages 21–40: 24 men, 41 women), 40 middle-aged (ages 41–64: 18 men, 22 women), and 48 older subjects (65+: 20 men, 28 women) and containing three races including 19 Asian, 113 Caucasian, and 21 Black or African American (Supplementary Figure S1A, B, Supplementary Table S1). Samples from different sexes in each assay were comparable in terms of age (old females: ∼76.1 vs old males: ∼76.2, *t*-test *p*-value = 0.97; young females:∼28.7 vs young males: ∼30.0, *t*-test *p*-value = 0.35). Then, we performed WGCNA analyses using expressed genes (n = 19,254) from 153 adult human PBMC samples (Supplementary Tables S2.1, S2.2). First, a soft thresholding power (*β* = 6) with a scale-free topology fitting index of *R* ^2^ > 0.80 was defined to establish an adjacency matrix (Supplementary Figure S2A). We used a tree-cutting algorithm to calculate the average linkage clustering, obtain gene co-expression modules, merge similar modules with a module eigengene over 0.75 (Supplementary Figure S2B), and gradually build a co-expression network (Figure 2A, Supplementary Table S3). After that, the co-expression genes were clustered into 22 modules with the size of the co-expression modules ranging from 68 to 3,891 genes and labeled with different colors (Figure 2B, Supplementary Table S3). To further quantify the correlation of genes in different modules, we calculated their eigengene adjacency on their correlation of the entire modules. Each module showed independent validation to each other, and higher correlation indicated higher co-expression interconnectedness (Figure 2B). Genes within the same module exhibited higher correlation than the genes between different modules.

In order to identify which factor mainly affected the variation in PBMC transcriptomes, the Pearson’s correlation coefficients between the 22 modules and the trait of age, gender, and race were calculated (Figure 2C, Supplementary Table S4). Notably, among them, green module significantly correlated with age positively (Pearson’s r = 0.39, *p* = 8.30e-07; Figure 2C), while darkolivegreen module showed the negative result (Pearson’s r = 0.45, *p* = 2.02e-08; Figure 2C). Moreover, sex and race individually showed a significant correlation in terms of the transcriptomic aging signatures. To investigate whether PBMC transcriptome changed gradually throughout the lifespan or rapidly at some stages, we analyzed various age brackets. Within each aging related module, we used the spline nonlinear regression for the transcriptomic profiles of the 153 samples in both green and darkolivegreen modules. Finally, our analyses indicated that PBMC transcriptome rapidly changed at two periods in adult lifespan: 1) a timepoint in early-forties, and 2) a later timepoint after 70 years of age (Figures 2D,E, Supplementary Table S5).

Besides, racial/ethnic differences in PBMC aging among adult lifespan were also important with profound effect on health. To determine the main causes of variation in transcriptomic data, we performed the principal component analyses (PCA) using expressed genes (n = 19,089) from high-quality samples. The first principal component (PC1) captured 14.9% of the variation in 153 microarray data and associated to age groups (Figure 2F, Supplementary Table S6). The variants of PC1 score between Asian and Caucasian samples were more significantly different, whereas the difference in Asian and Black or Africa American is not significant (Figure 2G). Besides, we used the genes in age-related modules (Pearson’s |r|≧0.19, *p*≦0.05, modules including green, darkolivegreen, sienna3, darkmagenta, greenyellow, brown, turquoise and red) to perform the PCA analysis. Similarly, the variants of PC1 score between Asian and Caucasian samples were also significantly different (Supplementary Figure S3A,B). Together, these results suggested that aging and race had both influenced the variation in PBMC transcriptomes.

### Aging-Related PBMC Transcriptome Dynamics in Asian (Chinese)

**FIGURE 3:**
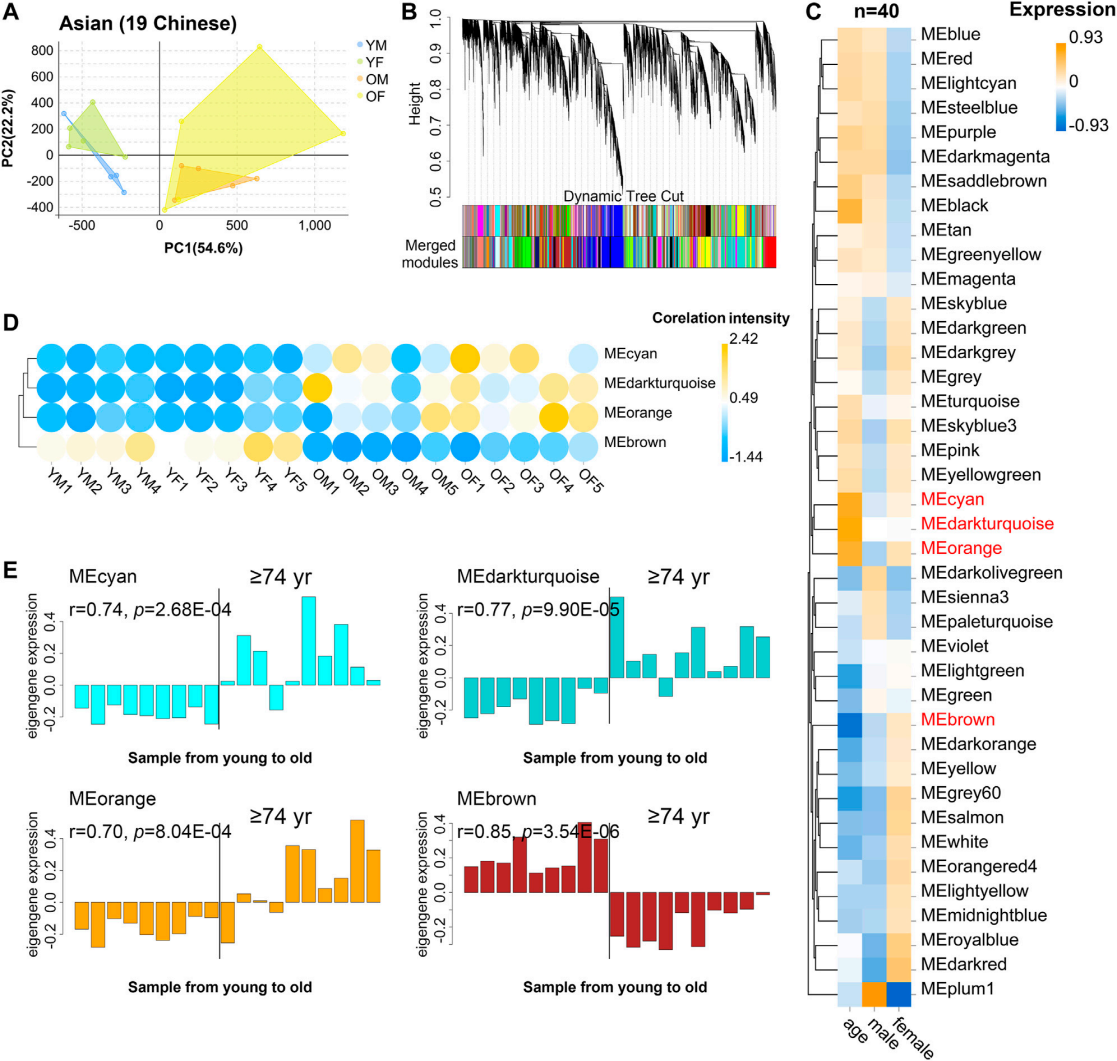
The characteristic gene expression of PBMC aging in Asian (Chinese). Transcriptome data of 19 healthy human subjects in Guangdong China were analyzed, and gene modules were constructed by WGCNA. **(A)** Principal component analyses. Young and old individuals were largely separated according to the principal component1 scores (PC1). **(B)** Cluster dendrogram. Each branch represents one gene, and each color below denotes one co-expression gene module. The two colored rows below the dendrogram represented the original and merged modules, respectively. **(C)** Heatmap of the Pearson’s correlation between trait (age and gender) and module eigengene (ME, *n* = 40). The column and row corresponded to trait and ME, respectively. The color in each cell represented corresponding correlation and scaled in the color legend. **(D)** Hierarchical cluster analyses of four interested modules. Based on the module-trait’s Pearson’s r and *p* value (absolute r > 0.5, *p* < 0.05), three modules (cyan, darkturquoise, and orange) showed relatively lower expression in young PBMC groups and high expression in the aged, while the brown modules showed the opposite result. Each dot represented an individual. **(E)** The histograms of the eigengene expression in the four age-related modules from young to old.

We recruited 19 community dwelling healthy volunteers (10 female, 9 male) whose ages span 21–93 years old (Supplementary Table S7): 9 young (age 21–30: 5 female, 9 male), and 10 old donors (age 74: 5 female, 5 male), from Guangdong Province of China. Samples from different sexes in each assay were comparable in terms of age (males: ∼81.4 vs females: ∼85.2; *t*-test *p*-value = 0.41), as well as the young samples (males: ∼24.5 vs females: ∼25.8; *t*-test *p*-value = 0.50). Then, PBMC transcriptome of these 19 donors was profiled using RNA-seq. As a result, 29,367 genes were selected after normalization of raw expression counts, excluding the genes with no expression in all samples (Supplementary Table S8). PCA of the 19 PBMC transcriptomes of Asian donors (Chinese) revealed that young and old samples were divided into two parts, and females had larger variants than males, especially in the old group (Figure 3A). To further identify the related genes of PBMC aging, WGCNA was conducted using FPKM values of 29,367genes (FPKM > 1 of all sequenced transcript) and the trait of age and sex (Figure 3B). Genes with the similar expression pattern were clustered into the same module to generate a cluster dendrogram (Figure 3B). The sample dendrogram and trait heatmap were visualized to understand the relationship between the corresponding gene expression data and biological traits (Figure 3C). Forty modules were obtained, of which four ME-modules (cyan, darkturquoise, orange, brown) showed significant correlations with age, with the absolute Pearson’s correlation coefficient |r| ≥ 0.70 (*p* < 0.01) (Figure 3C, Supplementary Table S9). Further, a consensus clustering also confirmed the four main modules were clearly separated between young to old (Figure 3D). Similarly, based on the module eigengene (ME) expression profile and the ages of the donors, these four significant modules all showed sharp changes at the age of 74 (Figure 3E, Supplementary Table S10). These results suggested these four gene modules were highly associated with chronological age in Asian (Chinese), especially for the brown (r = 0.85, *p* = 3.54E-06) and darkturquoise modules (r = 0.77, *p* = 9.90E-05).

### Novel and Known Age-Associated Genes and Pathways Associated With PBMC Aging in Asian (Chinese)

**FIGURE 4:**
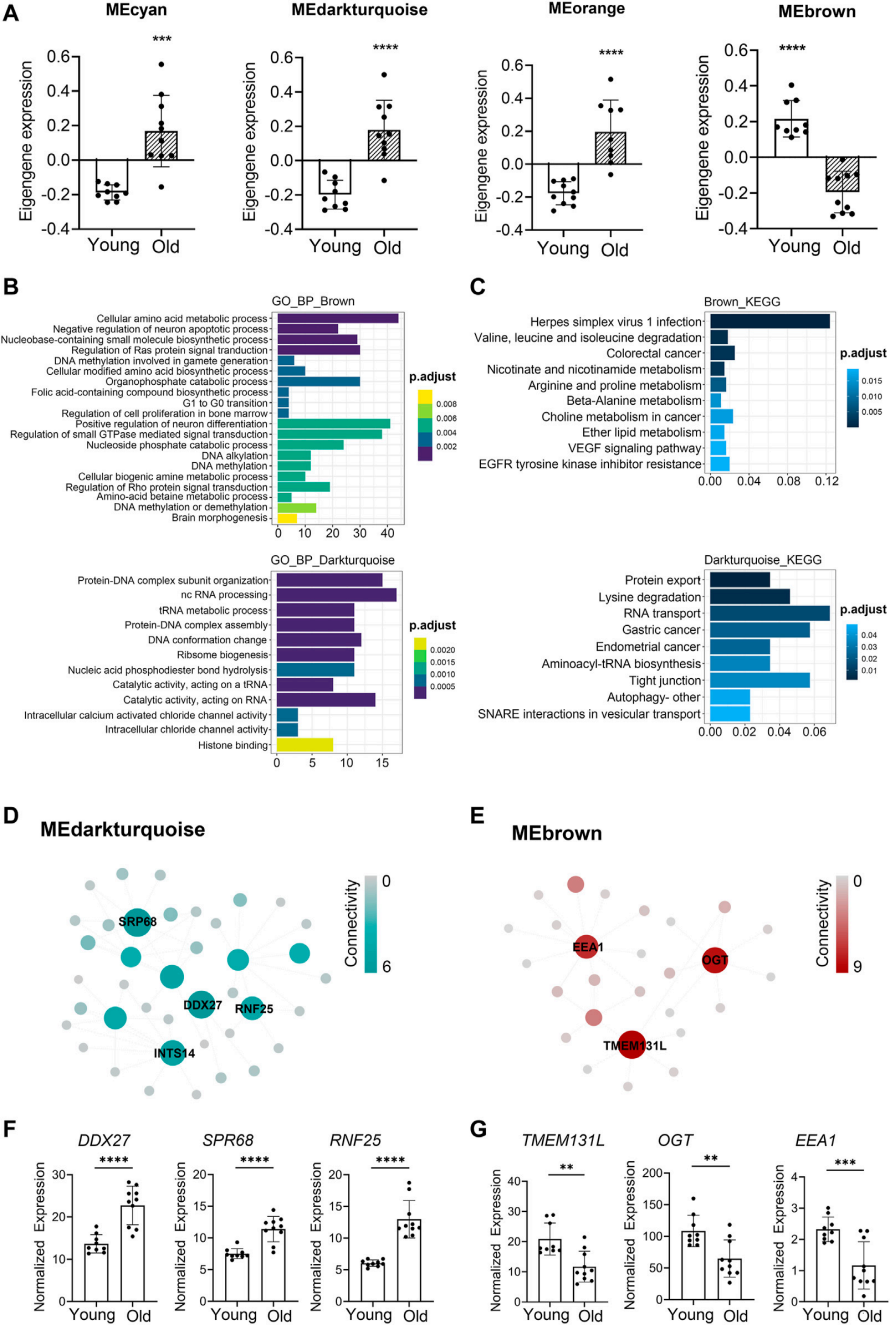
Novel and known age-associated genes and pathways associated with PBMC aging in Asian (Chinese). Gene Ontology (GO) and KEGG pathway enrichment analyses were conducted to analyze the biological functions of modules. Module eigengene (ME) was defined as the first principal component of the expression matrix of the corresponding module and was considered as a representation of the gene expression profiles in a module. **(A)** The transcriptomic expression of age related modules changed significantly among young and old individuals. Based on ME expression profile of the four interesting modules, the expression of cyan, darkturquoise, and orange modules was downregulated, while brown module showed the opposite results. Box plots represent mean ± standard deviation (SD). The *p*-value was calculated by the student’s t-test, *n* = 19, *p < 0.05, **p < 0.01, ***p < 0.001, ****p < 0.0001. **(B,C)** Top 10 GO biological process functional annotation **(B)** and KEGG pathway enrichment **(C)** analyses for the brown and darkturquoise modules. The Benjamini-Hochberg FDR multiple test correction was also applied to assess significance of hypergeometric *p*-values at a false discovery rate (FDR) of 5%. The color represented the adjusted *p*-values. **(D,E)** Hub gene detection for the darkturquoise **(D)** and brown **(E)** modules. PPI network of the brown and darkturquoise modules was based on the STRING database. Each node represented a protein-coding gene, and the size of each node was mapped to its connectivity (also known as degree). **(F,G)** The verification of the hub genes. The top of three genes in brown and darkturquoise modules were selected, and its mRNA abundance of these hub genes was detected in young and old individuals. Box plots represent mean±standard deviation (SD). The *p*-value was calculated by the student’s t-test, young = 9, old = 10, *p < 0.05, **p < 0.01, ***p < 0.001, ****p < 0.0001.

WGCNA analyses defined that the module eigengene (ME) was the first principal component of a given module and could be considered as a representative of the module’s gene expression profile. Based on ME expression profile of the four significant modules, the expression of cyan, darkturquoise, and orange modules was downregulated in young donors, while brown module showed the opposite results (Figure 4A). To further explore the biological functions of the most closely age-related modules (brown module Pearson’s r = 0.85, *p* = 3.54E-06; darkturquoise module Pearson’s r = 0.77, *p* = 9.90E-05), we performed Gene Ontology (GO) enrichment analyses, as well as pathway ontology analyses by using clusterProfiler R package (Yu et al., 2012) (Supplementary Table S11). The enrichment analyses revealed that in the brown module, the top two enriched terms in GO ontology were “Cellular amino acid metabolic process” (FDR = 5.74E-04) and “Negative regulation of neuron apoptotic process” (FDR = 8.43E-04) (Figure 4B); for the KEGG pathway analyses, the top enriched terms were “Herpes simplex virus 1 infection” (FDR = 9.19E-09) and “Valine, leucine and isoleucine degradation” (FDR = 1.33E-03) (Figure 4C). Meanwhile, functional annotations of darkturquoise module genes showed the top enriched terms in the GO databases were “Protein-DNA complex subunit organization” (FDR = 7.68E-07) and “ncRNA processing” (FDR = 1.33E-06) (Figure 4B). Moreover, genes in darkturquoise module were found to be significantly enriched in protein export and lysine degradation signaling pathway (Figure 4C). These findings together with previous research, which found persistent virus infections and metabolic dysregulation, were closely related with immune aging (Hamrick and Stranahan, 2020), implying that the above signaling pathways might play an important role in aging.

To identify key genes associated with chronological age, we performed a more detailed analyses of the brown and darkturquoise modules. First, a total of 924 differentially expressed genes (DEGs) in the 19 Chinese PBMC transcriptomic data were found to be dysregulated in old individuals (|logFC| ≥ 1 and adjusted *p* < 0.05). Then, as shown in Venn diagram Supplementary Figure S4, 278 and 19 overlapping genes were extracted from the brown module and darkturquoise module in 19 Chinese PBMC DEG dataset, respectively. Subsequently, the protein-protein interaction (PPI) network among the overlapping genes was established to identify potential aging related hub genes by using the STRING database. Then, based on the Maximal Clique Centrality (MCC) scores calculated by cytoscape, the top six highest-scored genes, including probable ATP-dependent RNA helicase (DDX27), Signal recognition particle subunit (SRP68), E3 ubiquitin-protein ligase (RNF25), Transmembrane protein 131-like (TMEM131L), UDP-N-acetylglucosamine--peptide N-acetylglucosaminyltransferase 110 kDa subunit (OGT), and Early endosome antigen 1 (EEA1), exhibiting the highest connections with other genes were identified for further investigation (Figures 4D,E). Strikingly, the mRNA abundance of these hub genes was significantly associated with chronological age (Figures 4F,G). It was previously demonstrated that TMEM131L could regulate immature single-positive thymocyte proliferation arrest by acting through mixed Wnt-dependent and -independent mechanisms (Maharzi et al., 2013). Reports also demonstrated O-GlcNAc transferase (OGT) level was decreased in multiple aged tissues and suggested that dysregulation of OGT related O-GlcNAc formation might play an important role in the development of age-related diseases (Fulop et al., 2008). Researchers also reported the abundance of EEA1 proteins was altered in the brains of aged mice (Ve et al., 2020). Moreover, SRP68 has been reported for its association with cellular senescence, while the ubiquitination-related genes RNF25 is not clear in immune aging. These data supported the notion that TMEM131L, OGT, EEA1, DDX27, SRP68, and RNF25 played important roles during PBMC aging, which might function as the novel candidate biomarkers of aging for Chinese individuals.

### Age-Related Transcriptional Variation of Caucasians

**FIGURE 5:**
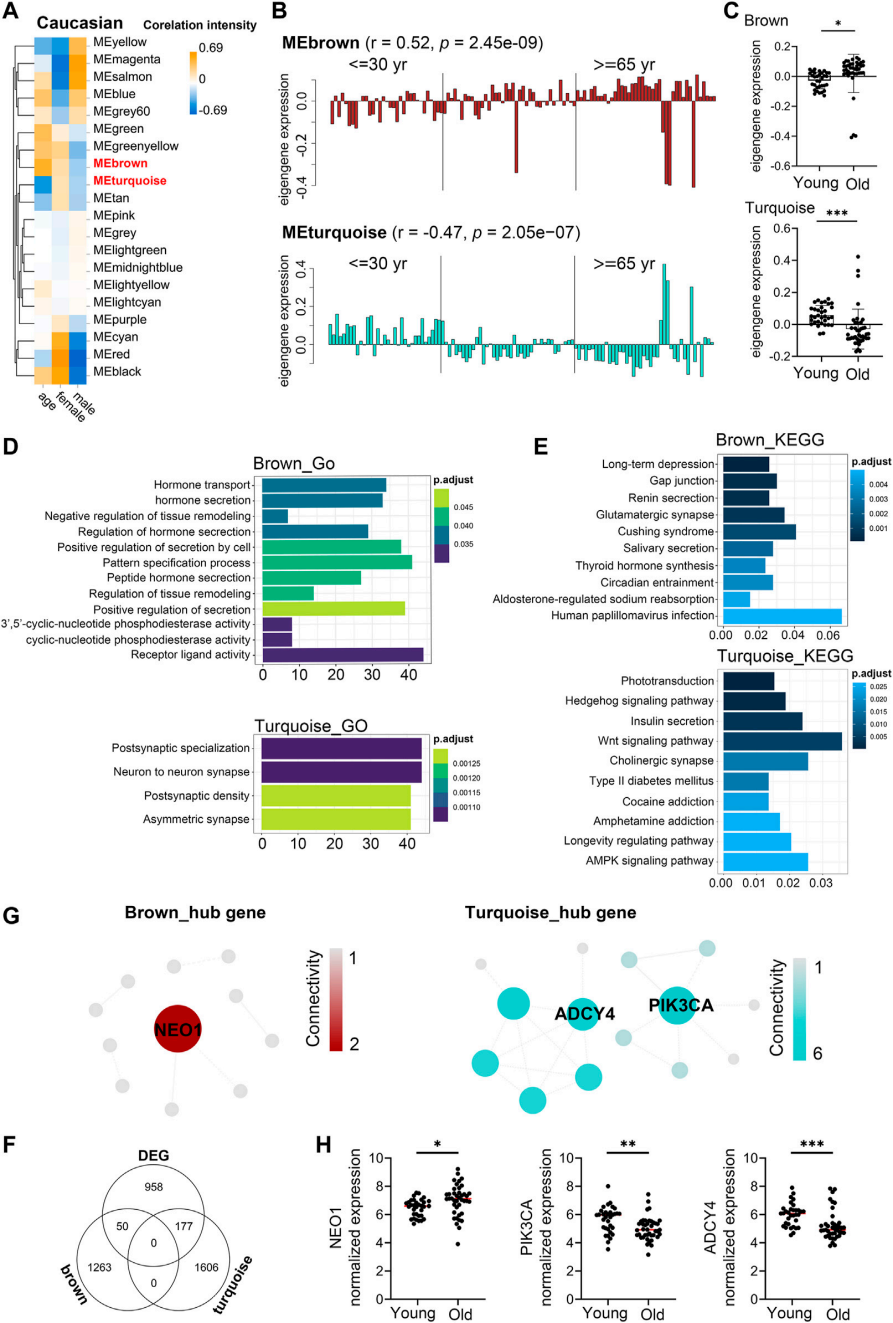
Aging-related changes in PBMC transcriptomes in Caucasian and its hub gene detection. Transcriptome data of 113 healthy Caucasian subjects in 10KIP were analyzed and gene modules were constructed by WGCNA. **(A)** Module-trait relationships. Row and column corresponded to module eigengenes and clinical trait (age and gender), respectively. Each cell contained the corresponding Pearson’s correlation coefficient and *p*-value. **(B)** The histograms presentation of the eigengene expression in the brown and turquoise modules from young to old. **(C)** Comparison of eigengene expression of the brown and turquoise modules between young and older. The transcriptomic expression of age related modules changed significantly among young and old individuals. Box plots represent mean ± standard deviation (SD). The *p*-value was calculated by the student’s t-test, n = 113, *p < 0.05, **p < 0.01, ***p < 0.001. **(D,E)** Gene Ontology (GO) and KEGG enrichment analyses for the genes in the brown and turquoise modules. The top 10 of the GO enriched biological process and enriched KEGG pathway were shown. The Benjamini-Hochberg FDR multiple test correction was also applied to assess significance of hypergeometric *p*-values at a false discovery rate (FDR) of 5% in GO and KEGG enrichment analyses. The color represented the adjusted *p*-values, and the size of the bars represented the gene number. **(F)** The Venn diagram showed the overlapping genes among differential expression genes (DEG) and co-expression modules. In total, 50 and 177 overlapping genes were listed in the intersection of DEG lists and two co-expression modules, respectively. **(G)** Hub gene detection for the brown and turquoise modules by using the STRING database. **(H)** The verification of the hub genes. The top of genes in brown and turquoise modules were selected, and mRNA abundance of these hub genes was detected in young and old individuals. Box plots represent mean ± standard deviation (SD). The *p*-value was calculated by the student’s t-test, young = 33, old = 41, *p < 0.05, **p < 0.01, ***p < 0.001, ****p < 0.0001.

Similarly, to investigate the aging-related gene modules in PBMC transcriptomes in Caucasian individuals, we performed WGCNA on microarray data from 113 Caucasian individuals, including 48 young (<40 years), 25 mid-age (40–65 years), and 41 health elderly (65–90 years). Old samples from different sexes in each assay were comparable in terms of age (males: ∼76.9 vs females: ∼76.8; *t*-test *p*-value = 0.97), as well as the young samples (males: ∼29.8 vs females: ∼28.6; *t*-test *p*-value = 0.43). Then, a total of 16,376 genes from these transcriptomic data were used for this computation. Twenty major gene modules (each module containing ≥160 genes) were identified. Similarly, we plotted the heatmap of module-trait relationships to evaluate the association between each module and the trait of age and sex (Figure 5A; Supplementary Table S12). The results revealed that the brown and turquoise module were found to have the highest association with chronological age (brown module: r = 0.52, *p* = 2.45e-09; turquoise module: r = −0.47, *p* = 2.05e−07). More interestingly, these two aging-related modules showed two periods in the human lifespan during which the PBMC gene expression underwent abrupt changes: 1) a timepoint in early thirties, and 2) a later timepoint after 65 years of age (Figures 5B,C). GO functional enrichment analyses suggested that the brown and turquoise modules were mainly enriched in hormone transport and postsynaptic specialization, respectively (Figure 5D, Supplementary Table S13). Moreover, KEGG pathway enrichment analyses showed that the genes of brown module were mainly categorized into long-term depression and gap junction, while the turquoise module was mainly enriched in phototransduction and hedgehog signaling pathway (Figure 5E). Next, we focused on the core genes of the brown and turquoise modules. By using the differential expression analyses, we identified 1,185 genes differentially expressed with chronological age in Caucasian, and 50 and 177 of these DEG genes were members of the brown and turquoise module, respectively (Figure 5F). Subsequently, the 50 and 177 genes from brown and turquoise module were subject to hub gene identification using the STRING database, respectively (Figure 5G). The results showed that the top two hub genes (Adenylate cyclase 4, ADCY4; Phosphatidylinositol 4,5-bisphosphate 3-kinase catalytic subunit alpha isoform, PIK3CA) in turquoise module were significantly downregulated in PBMCs of old adults (Figure 5H), whereas immunoglobulin superfamily DCC subclass member 2 (NEO1) from brown module showed the opposite result in the Caucasian cohorts (Figure 5H). From the aging atlas website (https://bigd.big.ac.cn/aging/age_related_genes), ATP Pyrophosphate-Lyase 4 (ADCY4) and Serine/Threonine Protein kinase (PIK3CA) have both involved in Longevity regulating pathway. As reported, ADCY4 catalyzed the formation of the signaling molecule cAMP in response to G-protein signaling (Ludwig and Seuwen, 2002), and PIK3CA participated in cellular signaling in response to various growth factors, which also involved in the activation of AKT1 upon stimulation by receptor tyrosine kinases ligands such as EGF, insulin, IGF1, VEGFA, and PDGF (Yamaguchi et al., 2011). Besides, Gulati *et al.* reported neogenin-1 (NEO1) was associated with the long-term HSCs (LT-HSCs) expand during age (Gulati et al., 2019). Taken together, these data also revealed that ADCY4, PIK3CA, and NEO1 were critical in aging, which might serve as the novel candidate biomarkers in Caucasian individuals during the PBMC aging.

### Shared Transcriptomic Signatures of Aging Between Asian (Chinese) and Caucasian

As age-expectation is ethnicity dependent (Menkin et al., 2017), we sought to test whether gene expression in PBMC of aging individuals differed across racial/ethnic groups. Since co-expressed genes may be co-regulated by the common transcription factors (TFs) and microRNAs (Kuleshov et al., 2016; Xie et al., 2021), we performed gene-set enrichment analysis using TRRUST and mirtarbase database (Han et al., 2018; Huang et al., 2020) for the brown module from Asian (Chinese) and the turquoise module from Caucasian, which were both negatively correlated with chronological age. The top significant enrichments of transcription factors PRDM1 and REST were observed in brown and turquoise respectively (Supplementary Figure S5). PRDM1 and REST were reported to be functionally associated with aging. For instance, PRDM1 in the brown module from Asian (Chinese) was found to be significantly correlated with age in human immune cells and might be involved in immunosenescence (Chi et al., 2019). REST had been reported regulated the human brain aging (Lu et al., 2014). Besides, the most enriched miRNAs were observed for miR 1931, miR501–3p, miR4738–5p, etc. Among them, miR501–3p had been reported as a potential biomarker related to the progression of Alzheimer’s disease (Hara et al., 2017). However, there were no evidences whether the expression of miR 1931, miR501–3p, and miR4738–5p changed with human immune aging.

**FIGURE 6:**
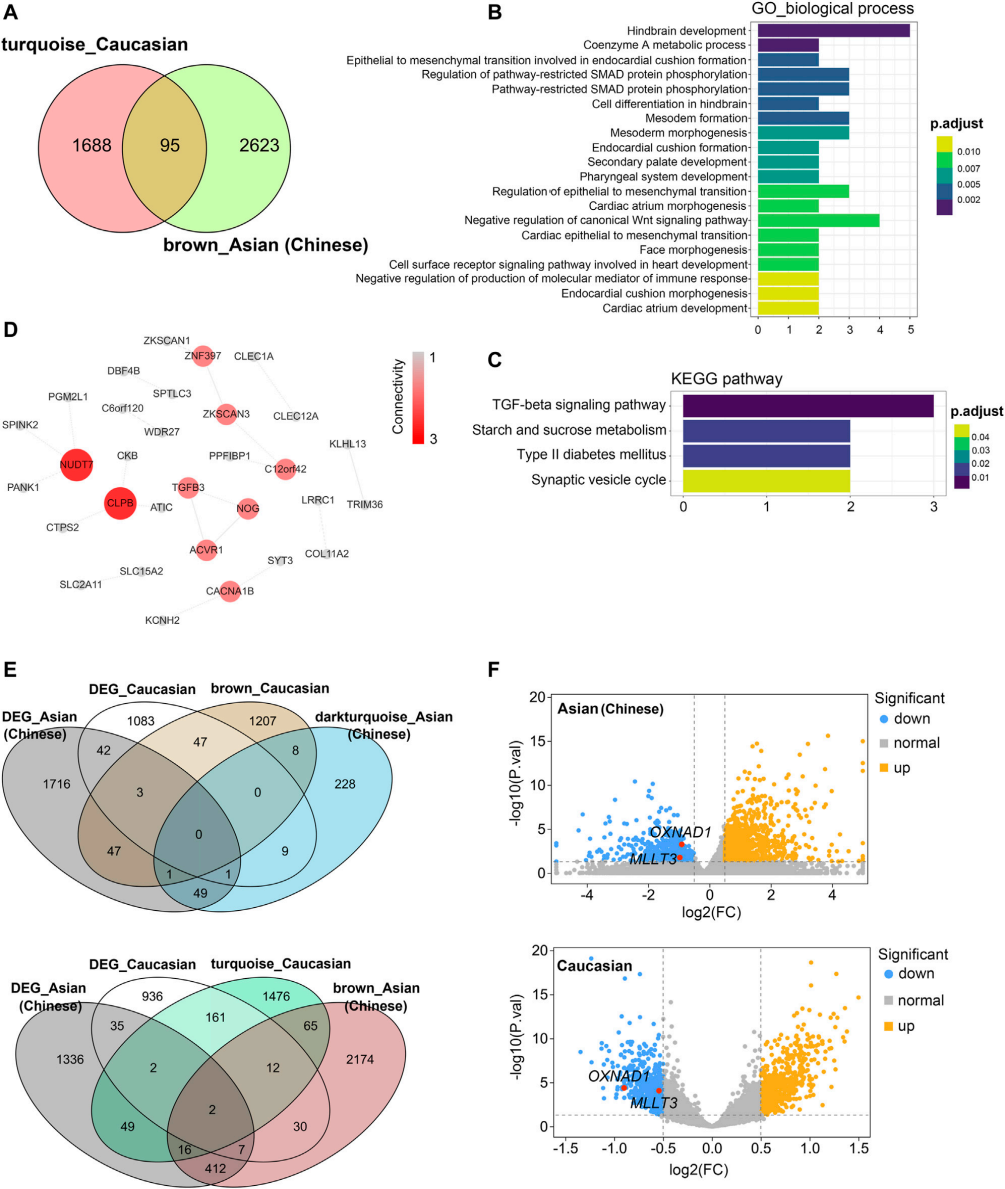
Shared transcriptomic signatures of PBMC aging between Caucasian and Asian (Chinese). The brown module from Asian (Chinese) and the turquoise module from Caucasian and its differentially expressed genes (DEGs) were compared and listed. **(A)** The Venn diagram of genes among turquoise module from Caucasian and brown module from Asian (Chinese). Despite thousands of race-specific gene associated with aging corresponding to 2,623 and 1,688 genes in Asian (Chinese) and Caucasian, 95 genes in Asian (Chinese) and Caucasian significantly overlapped. **(B,C)** GO and KEGG enrichment analyses for the 95 common shared genes. **(D)** Hub genes detection for 95 genes by using the STRING database, and visualized by the cytoscape. **(E)** The Venn diagram of differentially expressed genes (DEGs) with the age-related modules in White and Caucasian revealed two aging-specific gene markers. **(F)** The two overlapping genes (OXNAD1 and MLLT3) were both downregulated, as shown in the volcano diagram for the DEG genes in Asian and Caucasian dataset.

Then, we compared these two modules for common expressed genes. Ninety-five genes were shared between Asian (Chinese) and Caucasian, despite thousands of race-specific genes associated with aging (2,623 and 1,688 genes in Asian and Caucasian, respectively; Figure 6A). Functional annotation of these 95 shared genes revealed that they were highly enriched in the GO biological process of hindbrain development and coenzymeA metabolic process, as well as in the KEGG pathway of TGF-beta signaling (Figures 6B,C). To uncover potential regulators of common transcriptomic variation in Asian (Chinese) and Caucasian, we identified hub genes by using the STRING database. Accordingly, the top-scored genes, including peroxisomal coenzyme A diphosphatase (NUDT7) and caseinolytic peptidase B protein homolog (CLPB), were selected as the hub genes (Figure 6D). Meanwhile, two genes (OXNAD1 and MLLT3) that shared between Caucasian and Asian (Chinese) aging-related modules showed common differential expression (Figure 6E). These two potential aging-specific markers (OXNAD1 and MLLT3) were both downregulated in old Asian (Chinese) and Caucasian (Figure 6F). These data uncovered that despite the stark contrast between races in aging-related gene expression pattern, our analyses were able to highlight shared aging biomarkers with common functional enrichment in Asian (Chinese) and Caucasian.

### Validated Shared Genes Involved in PBMC Aging

**FIGURE 7:**
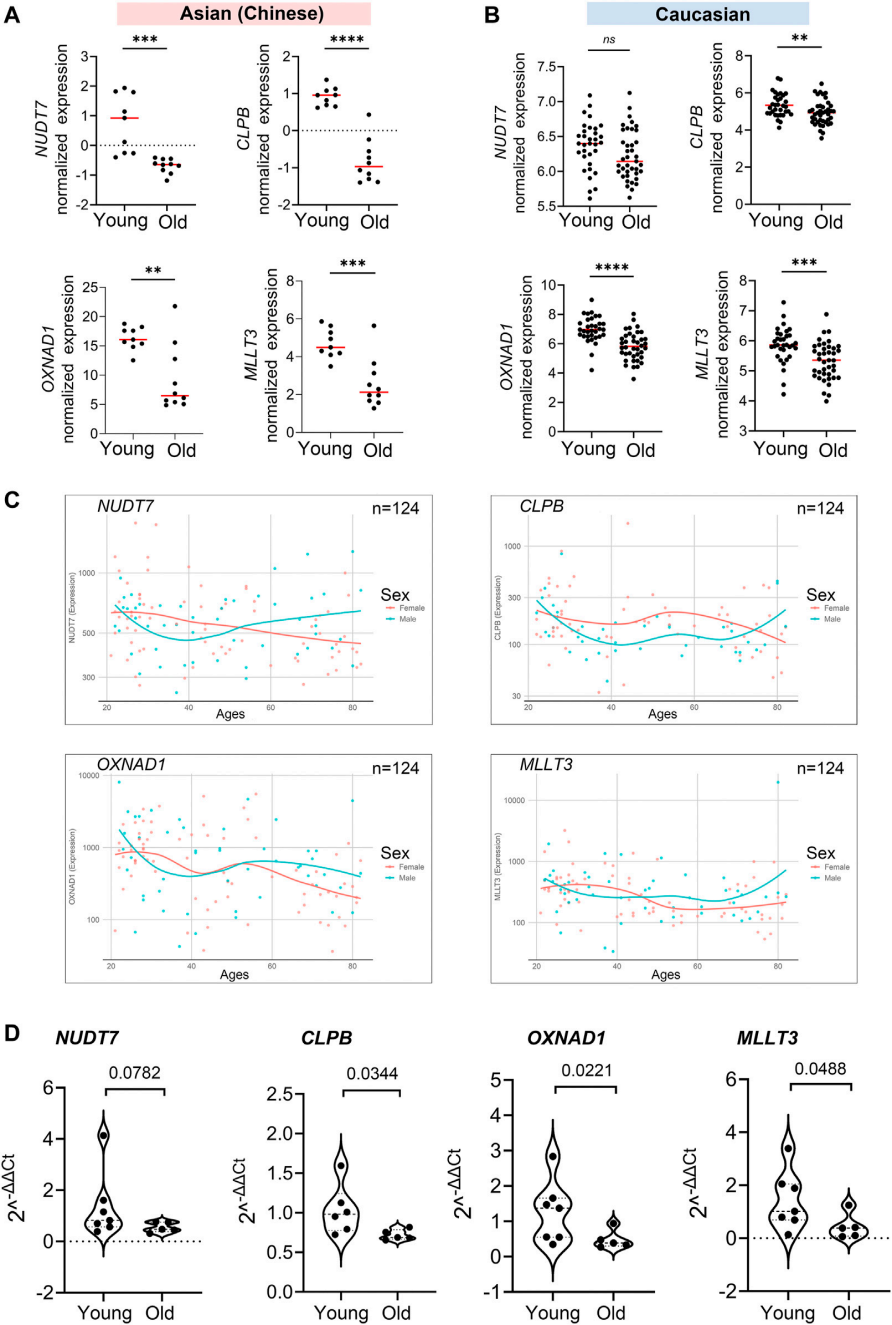
Validation of expression levels of the common hub genes involved in PBMC aging. The validation in Asian (Chinese) and Caucasian was performed using additional samples and 10KIP data, respectively. **(A,B)** Gene expression value of the hub genes among young and old samples in 19 Asian (Chinese) **(A)** and 153 Caucasian **(B)**. The data were expressed as the mean ± standard deviation (SD). Student’s t-test was used for statistical analyses. In Asian (Chinese), young *n* = 9, old *n* = 10; in Caucasian, young *n* = 33, old *n* = 40, *p < 0.05, **p < 0.01, ***p < 0.001, ****p < 0.0001. **(C)** Gene expression of hub genes among samples of man and woman during their lifespan, *n* = 124, including 19 Asian and 105 Caucasian. **(D)** Quantification of the four hub genes was confirmed and presented by the qPCR assay. The data were expressed as the mean ± standard deviation (SD). The *p*-value was calculated by the student’s t-test. Young *n* = 7; old *n* = 5, *p < 0.05, **p < 0.01.

After the 4 hub genes (*NUDT7*, *CLPB*, *OXNAD1*, and *MLLT3*) together shared in Asian (Chinese) and Caucasian, we verified the expression levels of the hub genes among young and older individuals using the public data, RNA-seq data, and qPCR assay. Interestingly, these 4 hub genes were significantly downregulated in old individuals compared with the youth in Asian (RNA-seq data from 19 Chinese, *n* = 19) and Caucasian (Microarray data from 10KIP, *n* = 89) (Figures 7A,B). They were also found to be downregulated in women during their lifespan in both Caucasian and Asian (*n* = 124) (Figure 7C). Meanwhile, in the public data of EGA (Id: EGAS00001002605), OXNAD1 and MLLT3 were significantly downregulated during their lifespan. NUDT7 showed a non-significant decreased expression trend compared to young individuals, while CLPB showed a non-significant increased expression (Supplementary Figure S6). To further investigate whether these 4 hub genes expressed differentially during PBMC aging, we collected samples from another 12 healthy volunteers residing in the Guangzhou, China, including 7 young adult (ages 21–30) and 5 aged healthy adults (ages 74+). We measured mRNA levels of these four hub genes (*NUDT7*, *CLPB*, *OXNAD1*, and *MLLT3*) in extracts of PBMC from the 12 subjects. Similarly, the mRNA level of NUDT7, CLPB, OXNAD1, and MLLT3 was both remarkably downregulated in the elderlies (Figure 7D). All the above-mentioned observations confirmed down-expression of OXNAD1 and MLLT3 is associated with PBMC aging in Asian (Chinese) and Caucasian.

## Discussion

Investigating how genes jointly preserve or change in different races during human PBMC aging is important, yet challenging. Recently, Peters *et al.* identified 1,497 differentially expressed genes with chronological age by meta-analysis in 14,983 individuals’ whole blood of European ancestry (Peters et al., 2015). However, gene expression difference between Asian and Caucasian during PBMC aging is still unclear. Therefore, our study aims to fill this gap in Asian and Caucasian subjects. Besides, WGCNA is an integrated bioinformatic analyses, which is characterized effectively and systematically to find modules and gene signatures highly related with the clinical trait, and provides a comprehensive characterization of the transcriptomic changes for disease’s functional interpretation (Langfelder and Horvath, 2008). Thus, in our study, several significant gene modules with the same expression trends were identified by using WGCNA integrated bioinformatic analyses in 19 Asian (Chinese) and 113 Caucasian populations. As suggested in functional annotation analyses by the clusterProfiler package, these module genes were mainly enriched in virus infection, amino acid metabolism, and differentiation, which were basic processes in aging mechanisms including dysregulation of herpes simplex virus 1 infection, valine, leucine, and isoleucine degradation, long-term depression, gap junction, and hedgehog signaling pathway. Furthermore, according to MCC scores from the CytoHubba plugin in Cytoscape, the top chronological age-related genes were screened out (namely, TMEM131L, OGT, EEA1, DDX27, SRP68, and RNF25 in Asian; ADCY4, PIK3CA, and NEO1 in Caucasian). According to reports in the literature, all of these genes were more or less closely associated with aging. Consistent with these reports, the expression of these genes was also found to be significantly regulated among young and old individuals in our study, supporting these genes might play a causal role in human PBMC aging. More importantly, our study revealed that although aging related transcriptomic alternations is a cumulative process throughout adult life, while there might exist two periods in the human lifespan during which the immune system underwent abrupt changes. The two breakpoints (30 and 65–70 years old) were much similar in Asian and Caucasian during their whole lifespan. A potential limitation of the two breakpoints is that we relied on a linear regression model to identify the time point which the immune system underwent abrupt changes. A recent study demonstrated that a quadratic regression model has a higher statistical fit to identify age-regulated expression trends in cross-sectional gene expression datasets (Gheorghe et al., 2014). So more complex models may be used to investigate the two breakpoints in future studies.

Despite well-characterized race differences in immune responses, disease susceptibility, and lifespan, it was unclear to what extend aging differentially affected peripheral blood cells of European and Asian ancestry. To fill this gap, we generated RNA-seq data in PBMCs from 19 age-matched healthy adults in Guangdong province of China and downloaded microarray data of 113 Caucasian PBMCs from 10 KIP (http://10kimmunomes.org/). By using WGCNA integrated bioinformatic analyses, we discovered a gene expression signature of aging that was shared between Asian (Chinese) and Caucasian ancestry including 1) 95 age-associated genes shared in 132 individuals and 2) four hub genes (NUDT7, CLPB, OXNAD1, and MLLT3) all decreased in old ages. According to reports in the literature about these four hub genes, NUDT7 acted as a coenzyme A (CoA) diphosphatase, which mediated the cleavage of CoA. NUDT7 functioned as a house-keeping enzyme by eliminating potentially toxic nucleotide metabolites, such as oxidized CoA from β-oxidation in the peroxisome, as well as nucleotide diphosphate derivatives, including NAD+, NADH, and ADP-ribose (Song et al., 2018). Furthermore downregulation of NUDT7 in mice accelerating senescence (Cho et al., 2003) was observed in the liver of starved mice (Bauer et al., 2004). Interestingly, OXNAD1, also known as oxidoreductase NAD-binding domain-containing protein, had been reported differentially expressed with chronological age (Peters et al., 2015). According to the uniprot annotation for CLPB, it might function as a regulatory atpase and be related to secretion/protein trafficking process, involved in mitochondrial-mediated antiviral innate immunity, and activated RIG-I-mediated signal transduction and production of IFNB1 and proinflammatory cytokine IL6 (Yoshinaka et al., 2019). Moreover, the hub gene of MLLT3 was a component of the superelongation complex and co-operated with DOT1L, which di/trimethylates H3K79 to promoted transcription (Steger et al., 2008; Li et al., 2014). Recently, Calvanese et al. found MLLT3 could govern human haematopoietic stem-cell self-renewal and engraftment (Calvanese et al., 2019). From above, NUDT7 and OXNAD1 both had an important role in cellular metabolism and aging, which was consistent with our finding of PBMC aging analyses, while the role of CLPB and MLLT3 in immune aging or senescence was unclear. Thus, by using co-expression networks, we identified new genes that were likely important in PBMC aging in Asian and Caucasian ancestry, opening new avenues of enquiry for future studies.

By WGCNA analyses, aging-specific regulatory modules and hub genes were identified in bulk PBMCs in Caucasian and Asian. Although this approach was effective in annotating the aging signatures, it was prone to biases in the differences of data quality and formats. Besides, as we had much smaller sample sizes for both PBMCs in Asian and the other ancestry groups, we used a nominal *p*-value threshold (*p* < 0.05) in these specific sub-analyses. Larger sample sizes will ultimately be needed to fully understand the transferability of the aging-transcriptome signatures. More importantly, further studies were needed to verify the important molecules, identified here (NUDT7, CLPB, OXNAD1, and MLLT3) as aging specific biomarkers of immune system aging. Future studies might be needed to describe these race differences at single-cell resolution and in sorted cells and to establish their functional implications. Taken together, these findings indicated that aging played a critical role in human immune system aging and should be taken into consideration while searching for molecular targets and time frames for interventions/therapies to target aging and age-related diseases.

## Data Availability

The datasets presented in this study can be found in online repositories. The names of the repository/repositories and accession number(s) can be found in the article/Supplementary Material.
