# Supplementary material for: Gene Expression Analysis Reveals Age and Ethnicity Signatures Between Young and Old Adults in Human PBMC
Source: Front Aging. 2022 Feb 3;2:797040. doi: 10.3389/fragi.2021.797040 (PMC9261324; doi:10.3389/fragi.2021.797040)
Supplement: Supplementary file 3 [file DataSheet2.docx]

**Gene Expression Analysis Reveals Age and Ethnicity Signatures Between Young and Old Adults in Human PBMC**

[Yang Hu](https://www.ncbi.nlm.nih.gov/pubmed/?term=Hu%20Y%5bAuthor%5d&cauthor=true&cauthor_uid=30210331)^1,2^, Yudai Xu^1^, Lipeng Mao^1,4^, Wen Lei^1^, Jan Jian Xiang^1^, Lijuan Gao^1^, Junxing Jiang^5^, Li`an Huang^6^, Oscar Junhong Luo^1,4^*, Jinhai Duan^3^*, Guobing Chen^1^*

1, Institute of Geriatric Immunology, Department of Microbiology and Immunology, School of Medicine, Jinan University, Guangzhou, Guangdong, China.

2, Guangdong Provincial Fertility Hospital, Guangzhou, Guangdong, China.

3, Eastern Department of Neurology of Guangdong General Hospital, Guangdong Academy of Medical Sciences, Guandong, China.

4, Department of Systems Biomedical Sciences, School of Medicine, Jinan University, Guangzhou, Guangdong, China.

5, Nanfang Hospital, Southern Medical University, Guangzhou, Guangdong, China.

6, Department of Neurology, the First Affiliated Hospital, Jinan University, Guangzhou, Guangdong, China.

*Correspondence should be addressed to: Drs. Oscar Junhong Luo, Jinhai Duan or Guobing Chen, Institute of Geriatric Immunology, School of Medicine, Jinan University, 601 Huangpu Avenue West, Guangzhou 510632, Guangdong Province, China. E-mails: luojh@jnu.edu.cn and [guobingchen@jnu.edu.cn](mailto:guobingchen@jnu.edu.cn).


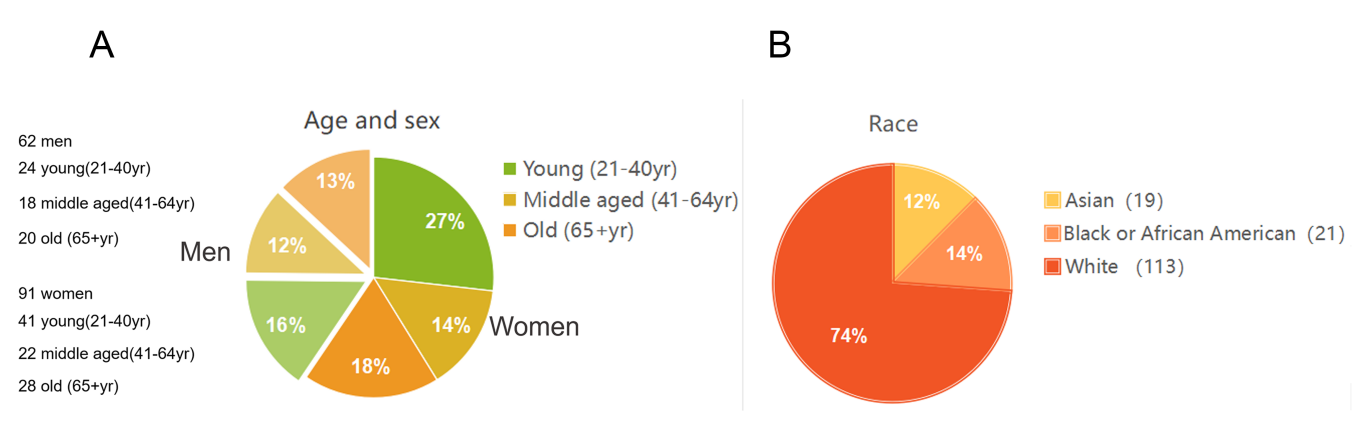


**Supplementary Figure S1. The age and race distribution of 153 individuals from 10 KIP dataset.** (A) The component of age among the 153 samples. (B) The component of race among 153 samples.


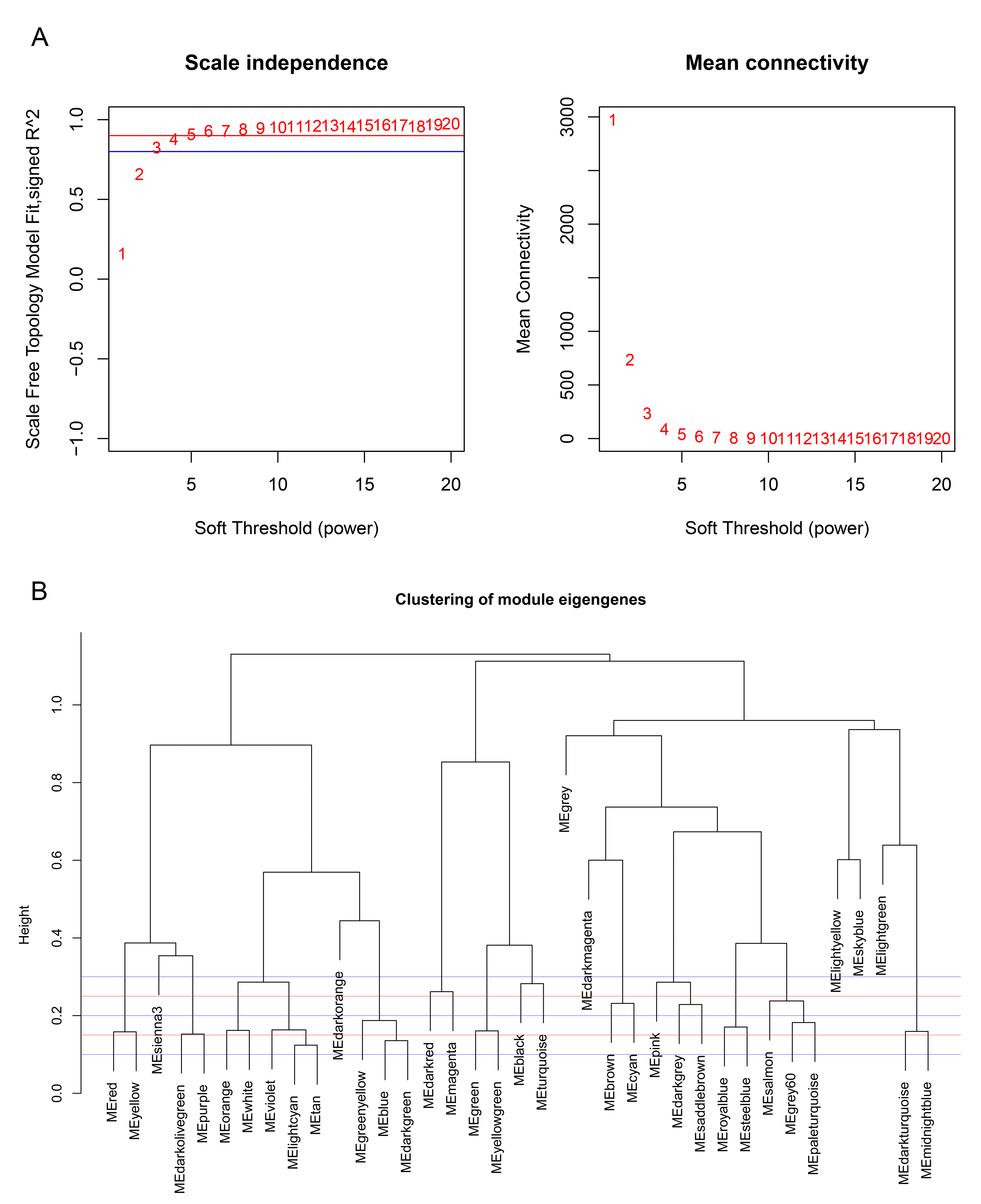


**Supplementary Figure S2. Power values and module clustering of WGCNA for the transcriptome data of 153 healthy human subjects in 10KIP.** (A) Selection of the soft-thresholding powers. The left panel showed the scale-free fit index versus soft-thresholding power. The right panel displayed the mean connectivity versus soft-thresholding power. Power 6 was chose for which the fit index curve flattens out upon reaching a high value (>0.9). (B) Meta-module identification. The module network dendrogram was constructed by clustering module eigengene distances. The horizontal line (blue and red line) represents the threshold (0.2) used for defining the meta-modules.


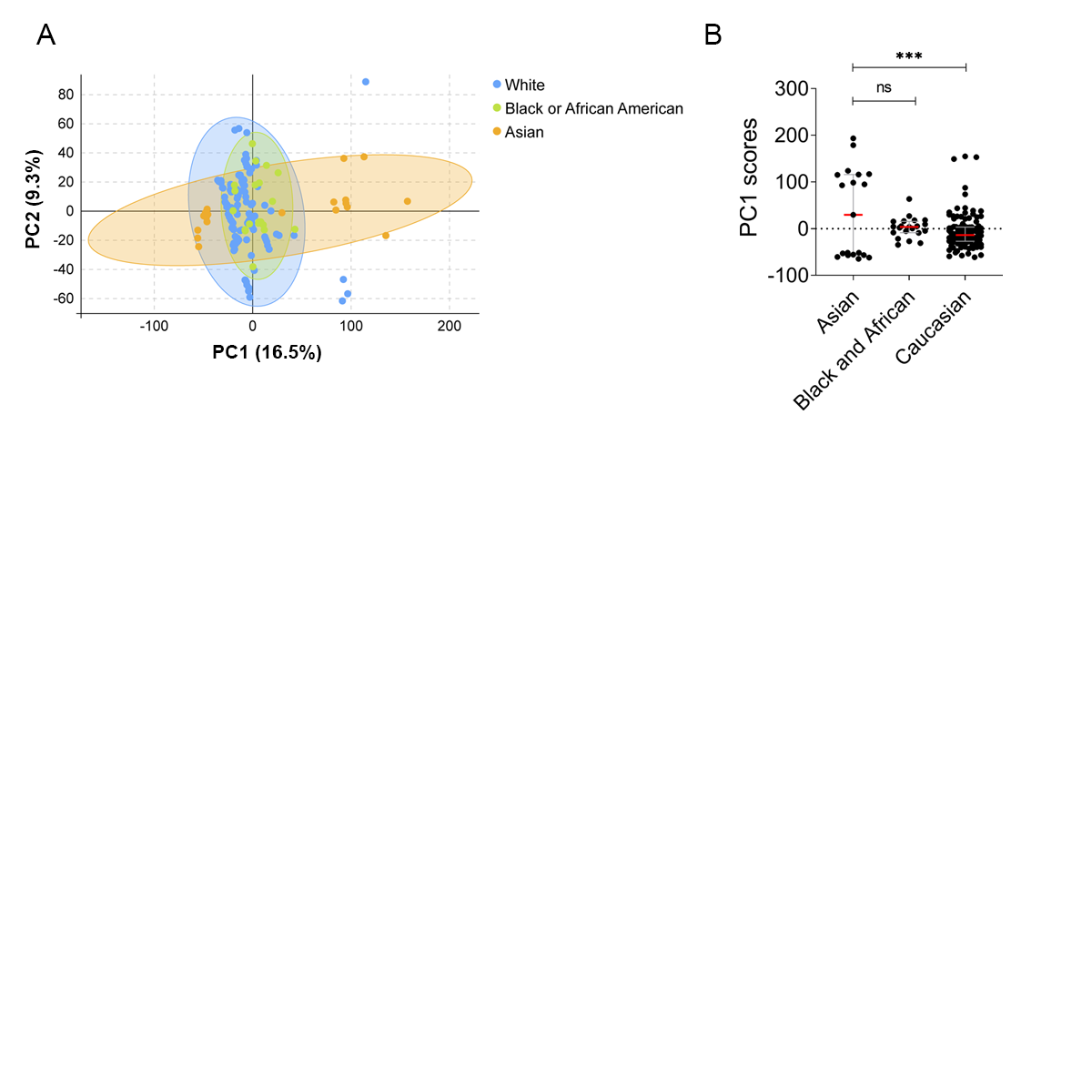


**Supplementary Figure S3. PCA analysis for age related gene modules in 153 individuals.** (A) Genes in 8 age-related modules (pearson’s r≧0.19, *p*≦0.05) were selected for PCA analysis in 153 individuals. (B) Principal component1 scores (PC1) were calculated for each individual from principal component analyses (PCA). PC1 scores from transcriptomic data were differentially expressed among different races. Wilcoxon rank-sum test was used to compare data from Asian (n = 19) and Caucasian (n = 113) or African American subjects. Dot plot represented median and IQR values; *****p* < 0.0001, ****p* < 0.001, ***p* < 0.01, **p* < 0.05, n.s.: non-significant.


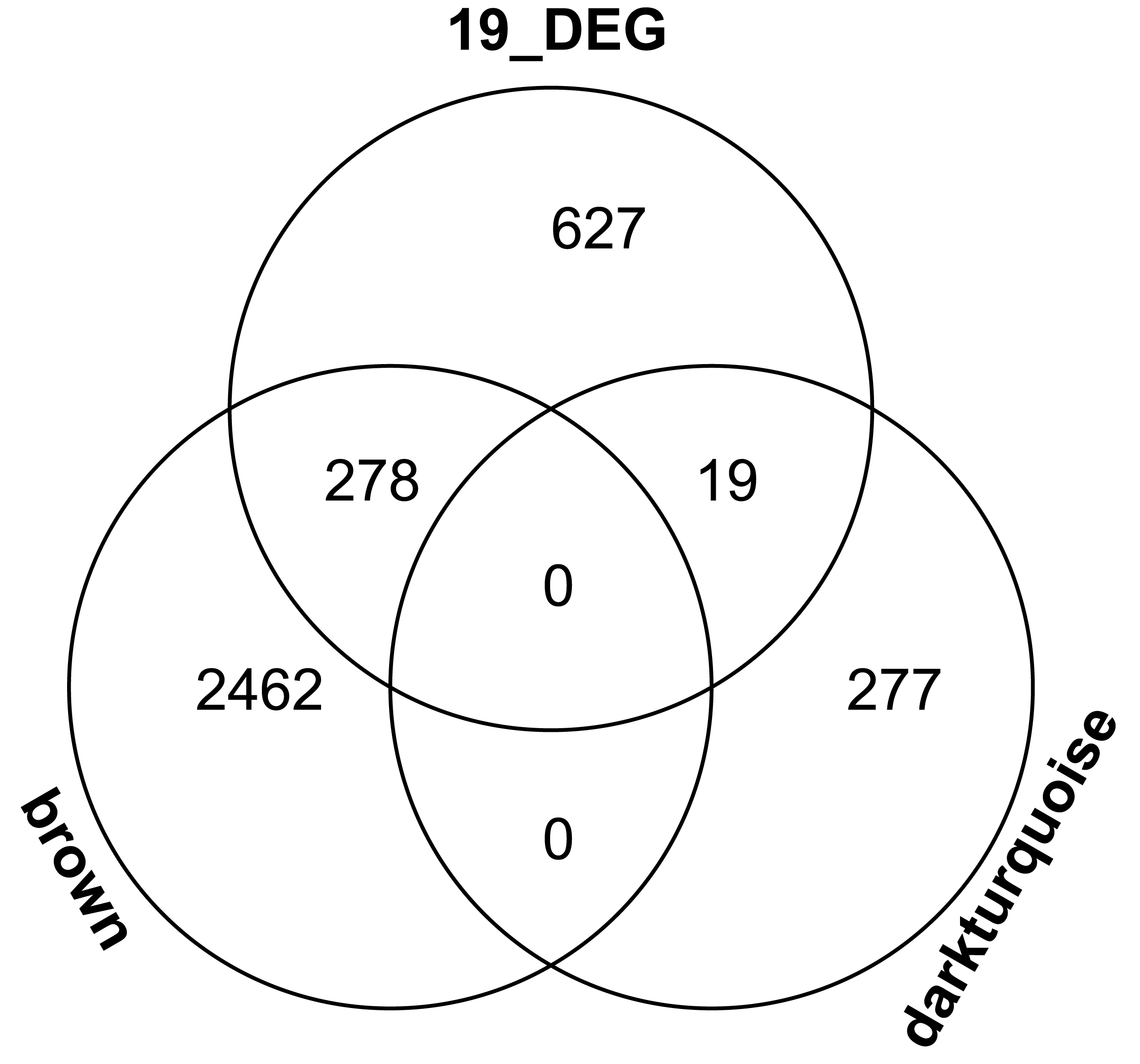


**Supplementary Figure S4. The venn diagram of genes among DEG lists and co-expression module in 19 Chinese RNA-seq data.** In total, 278 and 19 overlapping genes were listed in the intersection of DEG lists and two co-expression modules.


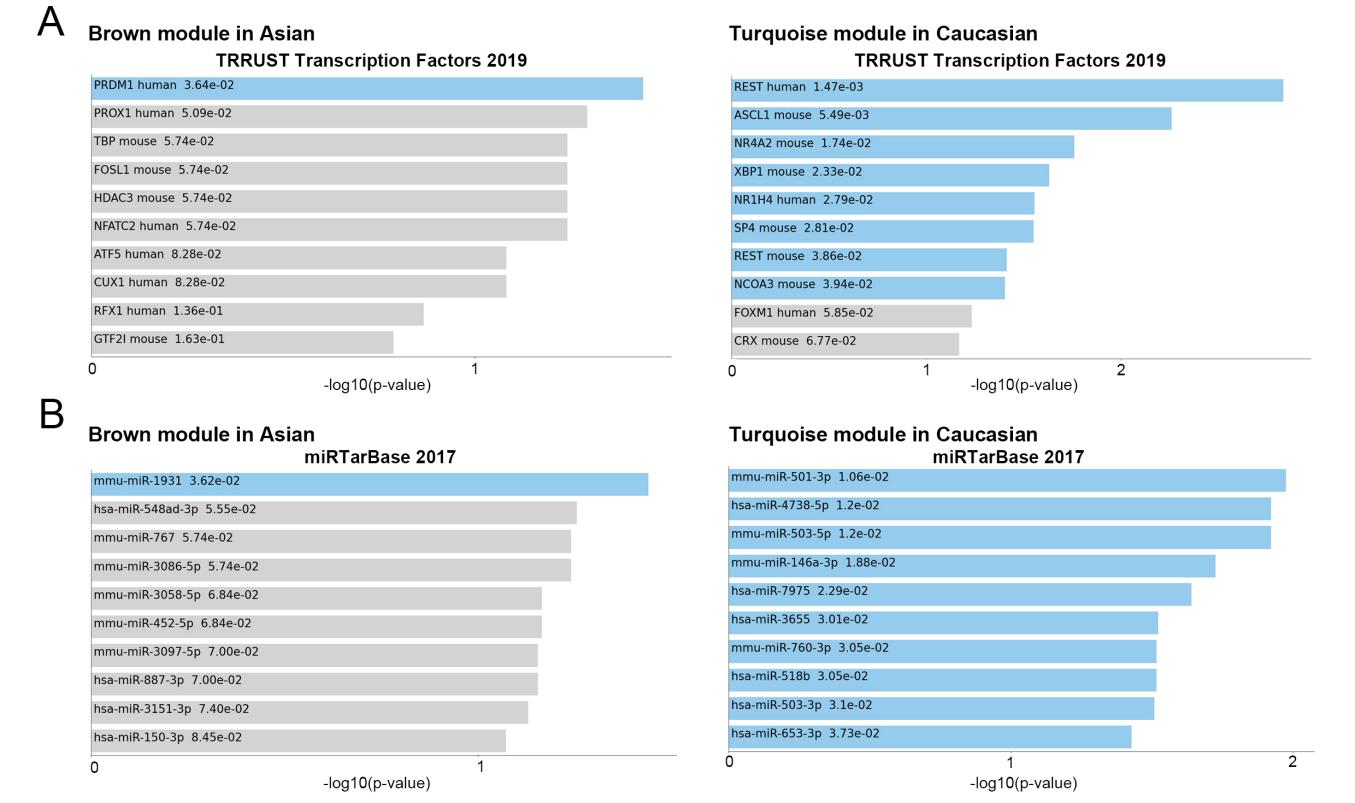


**Supplementary Figure S5. Potential factors regulating genes in Asian and Caucasian aging related module.** (A) Transcription factors and pathway. (B) Enriched seed and its associated microRNA.


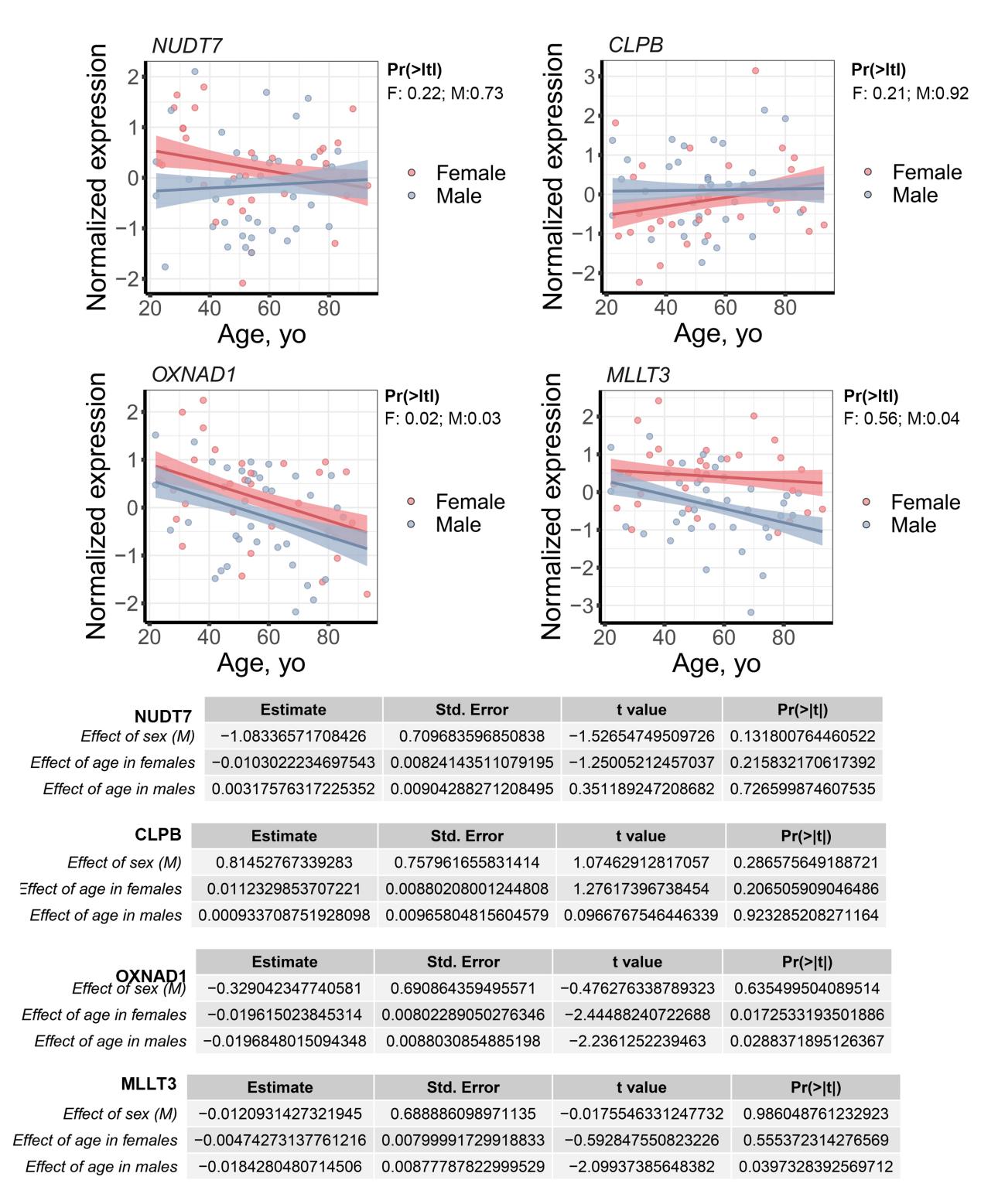


**Supplementary Figure S6.** **Validation of expression levels of the common shared hub genes involved in PBMC aging.** The validation in Asian (Chinese) and Caucasian were performed using the public RNA-seq data from EGA (Id: EGAS00001002605).
